# Correlated energy from radial density–energy relations

**DOI:** 10.1098/rsos.221402

**Published:** 2023-03-15

**Authors:** Adam L. Baskerville, Conor Gray, Hazel Cox

**Affiliations:** Department of Chemistry, School of Life Sciences, University of Sussex, Falmer, Brighton BN1 9QJ, UK

**Keywords:** energy–density relation, radial density, energy density, correlation energy

## Abstract

Here, we demonstrate that the radial distribution function can be mapped into a radial density–energy space and the relationship between the radial density and radial energy is linear for the ground and excited states of helium-like systems; the gradient of the resulting straight line delivers the energy of the state considered. To utilize this finding, a simple analytical expression for the total energy in terms of the density at the most probable nucleus–electron distance of the systems considered is derived using a fitting procedure.

## 1. Introduction

The study of few particle atoms and molecules, and even model systems such as the hookonium atom, remain a key tool in the arsenal of the quantum chemist. As the Schrödinger equation can only be solved exactly for a one-electron atom (or one-electron molecule within the Born-Oppenheimer approximation), it is necessary to make approximations. For few particle systems, it is possible to obtain high-accuracy results using various methods (see, e.g. [1–3]), which in some cases can be considered essentially numerically exact, e.g. Nakashima & Nakatsuji have reported the helium energy to over 40 digits [4,5]. These high accuracy wave functions can then be used to probe the interactions between fundamental particles, e.g. to provide new insights into electron correlation and Coulomb holes [6,7], the atom to molecule transition [8] and critical stability [9,10]. Furthermore, the study of few particle systems can play a key role in the development of approximations that can be extended to much larger systems: atoms, molecules, materials and beyond or as benchmarking data for new developments. For example, the ubiquitous LYP functional [11] used in density functional theory is based on a formula derived from the helium atom [12,13]. A key component in developing new approaches is the testing against model potentials and deriving simplified, preferably analytical, wave functions and properties that capture the essential physics of correlation between electrons whilst being relatively simply to handle computationally, see, e.g. Singh *et al.* [14].

In that vein, this article aims to demonstrate a simple relation between the radial energy and radial density that can be used to express the energy of a two-electron system in terms of the radial distribution function (RDF) at a single radial distance. Initially, we consider the hydrogen atom. Although perhaps it is a trivial observation that the radial energy is proportional to the radial density given that the wave function is an eigenfunction of the Hamiltonian, to our knowledge, a simple formula relating the energy to the RDF at a single radial distance has not been previously presented. Moreover, we show that the linear relationship between the radial density and radial energy holds for the helium atom, for both the singlet ground state and the triplet excited state. Given that the relationship is linear, by fitting to a subset of helium-like systems, it is possible to write an energy expression for any helium-like system as a function of the density at a *single* radial distance. Atomic units (a.u.) are used throughout, where $m_{e}=\hbar ={\left(4\pi \epsilon_{0}\right)}^{-1}=e=1$. The atomic unit of energy is the Hartree, *E*_*h*_, and the atomic unit of length is the Bohr.

## 2. Method

The Schrödinger equation for a three-body system, after separating off the centre of mass and taking the origin to be at particle 3, has the form:

2.1 $$\left(-\frac{1}{2\mu_{1}}\nabla_{1}^{2}-\frac{1}{2\mu_{2}}\nabla_{2}^{2}-\frac{1}{m_{3}}\nabla_{1}\cdot \nabla_{2}+\frac{Z_{1}Z_{3}}{r_{1}}+\frac{Z_{2}Z_{3}}{r_{2}}+\frac{Z_{1}Z_{2}}{r_{12}}\right)\psi =E\psi ,$$

where $\mu_{i}^{-1}=m_{i}^{-1}+m_{3}^{-1}$, *i* = 1, 2; the *m*_*i*_ and *Z*_*i*_ are the masses and charges of the particles; and *r*_1_ and *r*_2_ are the distances of particles 1 and 2, respectively, with respect to the third particle as the origin. In the case of an atom, and assuming *m*_3_ is the mass of the nucleus with *m*_3_ ≫ *m*_1_, *m*_2_, the effects introduced by locating the origin at particle 3 are very small. In this work, (2.1) is recast in perimetric coordinates, linear combinations of the inter-particle distances, i.e. *z*_1_ = (*r*_2_ + *r*_12_ − *r*_1_), *z*_2_ = (*r*_12_ + *r*_1_ − *r*_2_) and *z*_3_ = (*r*_1_ + *r*_2_ − *r*_12_), where *r*_1_ and *r*_2_ correspond to the electron–nucleus distances and *r*_12_ the electron–electron distance. A wave function of the following form is used:

2.2 $$\begin{array}{rl} \Psi (z_{1},z_{2},z_{3}) & =e^{-(1/2)(\alpha z_{1}+\alpha z_{2}+2\alpha z_{3})}\sum_{l,m,n=0}^{\infty }A(l,m,n)\{L_{l}(\alpha z_{1})L_{m}(\alpha z_{2})\pm L_{m}(\alpha z_{1})L_{l}(\alpha z_{2})\}L_{n}(2\alpha z_{3}), \end{array}$$

where *L*_*n*_ (*x*) is a normalized Laguerre polynomial of degree *n* and *α* is a nonlinear variational parameter. To satisfy the Pauli principle, in (2.2) the plus sign corresponds to the symmetric form of the wavefunction required for the singlet state and the minus sign corresponds to the antisymmetric form of the wavefunction required for the triplet state. The wave function is substituted into (2.1) which results in a 57-term recursion relation between the coefficients *A*(*l*, *m*, *n*) [15]. The infinite secular problem can then be written in the form of a generalized eigenvalue problem and solved in truncated form [1,15].

### 2.1. Radial distribution functions

According to the probabilistic interpretation of the wave function, the probability density is given by the square of the wave function. For an *N*-electron system, where the coordinate **x**_*i*_ corresponds to the space coordinates **r**_*i*_ and spin coordinate *s*_*i*_ of electron *i*, the electron density is defined as follows [16]:

2.3 $$\rho (r)=N\int \cdots \int |\Psi (x_{1},x_{2},\ldots ,x_{N}){|}^{2}\;ds_{1}\;dx_{2}\ldots \;dx_{N},$$

which represents the number of electrons per unit volume in a given state, and has atomic units of electrons bohr^−3^. The factor *N* appears as electrons are indistinguishable; therefore, the probability of finding any electron in volume element d**r**_1_ with arbitrary spin is *N* times the probability for one electron. In general, the probability density characterizes the spatial distribution of particles separated by the vector **r**, i.e.

2.4 $$\rho (r)=\left\langle \psi |\delta (r_{1}-r)|\psi \right\rangle ,$$

where **r**_1_ = **x**_1_ − **x**_*P*_ is the separation between particle 1 and some fixed point *P*, e.g. the centre of mass, or the nucleus or another particle. For spherically symmetric states, *ρ*(**r**) depends only on the lengths *r*_1_ = |**r**_1_| and so we can write as follows:

2.5 $$\rho (r)=\left\langle \psi |\delta (r_{1}-r)|\psi \right\rangle .$$

The radial density [17], usually referred to as the RDF, is defined as follows [17,18]:

2.6 $$D(r)=4\pi r^{2}\rho (r),$$

which represents the probability per unit length that the electron is found at a distance *r* from the nucleus, and in this work, it is normalized to one. For hydrogen-like systems, the RDFs are calculated using the following form:

2.7 $$D(r)=4\pi r^{2}|\psi (r){|}^{2},$$

for a range of discrete *r* values, where *r* is the nucleus–electron distance. For two-electron helium-like systems, the RDF for a spherically symmetric state in inter-particle coordinates is

2.8 $$\begin{array}{rl} D(r) & =4\pi r^{2}\left\langle \psi |\delta (r_{1}-r)|\psi \right\rangle =4\pi r^{2}\int_{0}^{\infty }\int_{\left|r-r_{2}\right|}^{\left|r+r_{2}\right|}|\psi (r,r_{2},r_{12}){|}^{2}\left(\frac{2\pi r_{2}r_{12}}{r}\right)\;dr_{12}\;dr_{2}, \end{array}$$

where 8*π*^2^*r*_1_*r*_2_*r*_12_ d*r*_1_d*r*_2_d*r*_12_ is the volume element before the Dirac delta function takes effect. In order to calculate this numerical integral to high accuracy, it was found that transforming to perimetric coordinates provided a faster and more robust method by simplifying the inner integral range. In perimetric coordinates, it has the following form (see electronic supplementary material):

2.9 $$D(r)=4\pi r^{2}\int_{0}^{\infty }\int_{0}^{2r}|\Psi (z_{1},z_{2},z_{3}){|}^{2}{\frac{\pi (z_{1}+z_{3})(z_{1}+z_{2})}{z_{2}+z_{3}}|}_{z_{2}=2r-z_{3}}\;dz_{3}\;dz_{1}.$$

The integrand in equation (2.9) is first simplified using the computer algebra system Maple [19], and then the output is passed to a highly optimized and parallelized C++ routine which is used to perform the numerical integration using the iterative, adaptive cuhre algorithm from the CUBA library [20]. All calculations are performed at 32-digit, with quadruple precision implemented using the DoubleDouble data type from the qd library [21].

### 2.2. Radial energy

Analogous to the RDF, we calculate the radial energy distribution by evaluating the expectation value of the Hamiltonian operator for discrete values of *r* value, i.e.

2.10 $$E(r)=\langle \Psi |\hat{H}|\Psi \rangle {|}_{r_{1}=r},$$

where the relevant one- or two-electron Hamiltonian operator is used. These ‘radial energies’, sometimes referred to as energy densities in the literature, are calculated using a procedure similar to that outlined for the RDF calculations, whereby a Maple program is used to simplify the integrand after substituting each value of *r*, and C++ is used to conduct the numerical integration using the cuhre algorithm at quadruple precision. It should be noted that there can be ambiguity in the definition of energy densities [22]. In the present work, *E*(*r*) is calculated using the ‘nabla squared’ ($\nabla^{2}$) rather than the ‘del dot del’ (∇⋅∇) definition of the local kinetic energy density.

### 2.3. Function fitting

Function fits are calculated by minimizing the least square difference between the set of data points, e.g. the end points of the lines in the radial density–energy space and the selected functional form, where the weighting coefficients are varied using the Levenberg–Marquardt algorithm [23].

## 3. Results

### 3.1. Hydrogen-like systems

For the ground state of a hydrogen-like atom, the RDF *D*(*r*), radial energy *E*(*r*) and the most probable distance from the nucleus *r*_max_, are given by

3.1 $$D(r)=4Z^{3}r^{2}\;e^{-2Zr},\;E(r)=-2Z^{5}r^{2}\;e^{-2Zr}\;\text{and}\;r_{\max}=\frac{1}{Z}.$$

**Figure 1.**
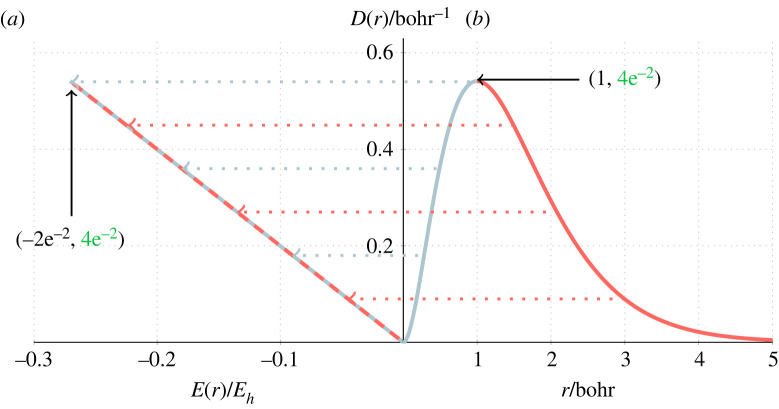
(*a*) The energy at *r* versus the radial density *D*(*r*) and (*b*) the RDF of the hydrogen atom. The dotted lines highlight that equal density values in the RDF have equal energy; mapping to the same point in the density–energy space.

Figure 1 shows the RDF for the hydrogen atom (*b*) and elucidates the relationship between *E*(*r*) and *D*(*r*), which we refer to as the radial density–energy space (*a*). For each value of the radial distance *r*, with radial density *D*(*r*), we calculate *E*(*r*). The end point of the line corresponds to the maximum in the RDF and has minimum energy as highlighted in figure 1, and the gradient of the radial density–energy line, *E*(*r*)/*D*(*r*), corresponds to the total ground state energy, *E*.

**Figure 2.**
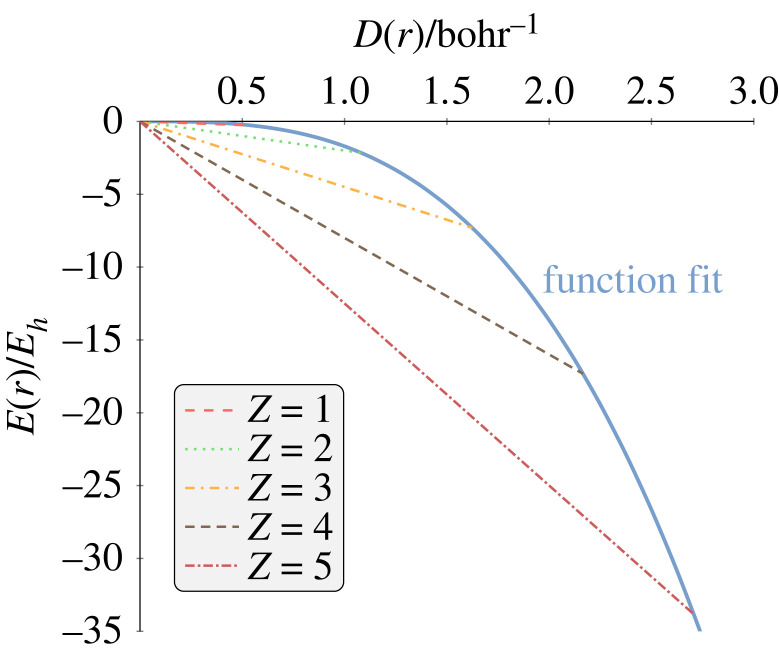
The radial energy as a function of the radial density for hydrogen-like systems. The solid blue line represents the fitting function, equation (3.2).

The RDF, and hence the radial density–energy relation, is dependent on the nuclear charge *Z* as shown in figure 2. The coordinates (*D*(*r*_max_), *E*(*r*_max_)) for a given *Z* are of the general form (4 *Z* e^−2^, − 2 *Z*^3^ e^−2^). Given that for any system, *E*(*r*) = *D*(*r*) = 0 as *r* → 0, the total energy *E* is simply *E*(*r*) divided by *D*(*r*), and thus, the total energy *E* can be determined from the radial energy and radial density at a single radius *r*. Figure 2 also demonstrates that a smooth curve connects the end points (*D*(*r*_max_), *E*(*r*_max_)) of each line for a given *Z*, which can be shown (see electronic supplementary material) to have the following form:

3.2 $$E(r_{\max})=-\frac{e^{4}}{32}D{\left(r_{\max}\right)}^{3},$$

i.e. *E*(*r*) can be determined from the radial density *D*(*r*). Furthermore, given that the energy is simply the gradient and choosing the non-zero value of *r* to equal *r*_max_, the total energy *E* in terms of *D*(*r*) is given as follows:

3.3 $$E=\frac{E(r)}{D(r)}=-\frac{e^{4}}{32}D{\left(r_{\max}\right)}^{2},$$

an analytical, closed-form expression that allows the calculation of the total energy of a one-electron system with knowledge of the radial electron density at just a single nucleus–electron distance, *r*_max_. It should be noted that (3.3) can be derived directly, without going via the radial energy, by simply writing the exponential parameter for the wave function decay in terms of the exact energy (see electronic supplementary material). However, this discussion is to show that it is possible to derive the relation using a fitting procedure that we will later apply to correlated systems where the exact form of the energy is unknown.

**Figure 3.**
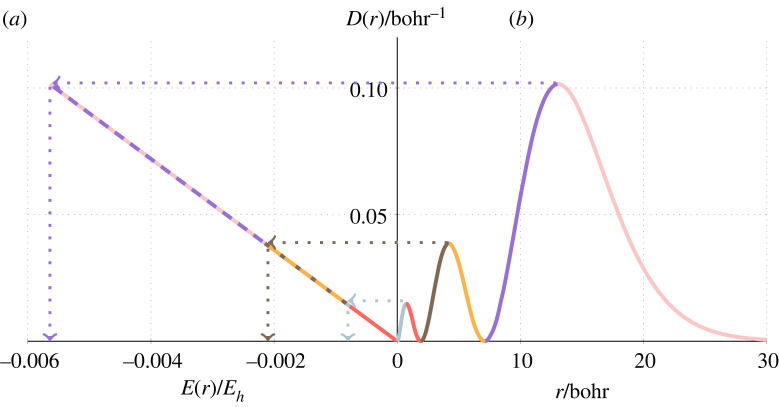
(*a*) *E*(*r*) versus the radial density *D*(*r*) and (*b*) the RDF of the 3 ^2^S state of the hydrogen atom. The dotted lines highlight the energy at each peak.

A similar relation can be found for excited states of the hydrogen atom. For example, figure 3 shows that the multiple peaks of the RDF of the 3 ^2^S state of hydrogen can also be collapsed to a single line in the density–energy space, and that the gradient of the line corresponds to the total energy (e.g. *E* = *E*(*r*_max_)/*D*(*r*_max_) = −0.0564/0.1015 = −0.0555 *E*_*h*_). By considering the RDF of a hydrogen atom with an electron in a 2*p*, 3*p* or 3*d* orbital, it is shown that this is a general feature (see electronic supplementary material). Plotting the radial energy of the 3 ^2^S state as a function of the radial density for a range of *Z* values results in a series of increasingly steeper lines in the radial density–energy space. A function that fits through the end point of the line for each *Z* value has a similar form to the ground state, i.e.

3.4 $$E(r_{\max})=-5.388\;921\;297\;837\;847D{\left(r_{\max}\right)}^{3},$$

where the precision of the constant pre-factor determines the accuracy of the energy. In addition, by considering the average of the three peak positions in the RDF for the 3 ^2^S state, $\bar{r_{\max}}$, and fitting a function to the curve that passes through the points $\left(D(\bar{r_{\max}}),E(\bar{r_{\max}})\right)$ for each *Z*, the pre-factor can be written as an exact analytical expression, i.e.

3.5 $$E(\bar{r_{\max}})=-\frac{81\;e^{8}}{512}D{\left(\bar{r_{\max}}\right)}^{3}.$$

Similar forms were found for other excited S states, which differ only in the pre-factor. Dividing equation (3.5) by the radial density results in an expression for the total energy of the excited state in terms of the radial density at a single radius, i.e. for 3 ^2^S states

3.6 $$E_{n=3}=-\frac{81\;e^{8}}{512}D{\left(\bar{r_{\max}}\right)}^{2}.$$

For example, for the hydrogen atom, the sum of the peak positions in the 3 ^2^S state is 18 (and in fact, the sum of the peak positions for the *n*th S state of a hydrogen atom corresponds to a pentagonal pyramid number), and thus, $\bar{r_{\max}}=6$. Calculating *E*(6) and *D*(6) and taking the quotient give the energy of the 3 ^2^S state.

### 3.2. Helium-like systems

#### 3.2.1. The 1 ^1^S state of helium-like systems

**Figure 4.**
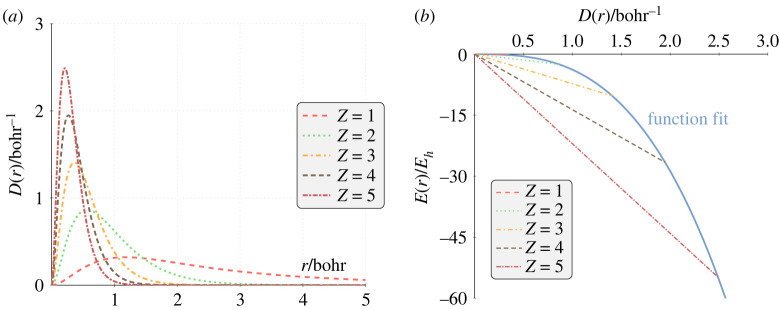
For helium-like systems: (*a*) RDFs and (*b*) radial density–energy profiles.

The RDFs for two-electron helium-like systems with infinite nuclear mass (i.e. where *m*_3_ = ∞ in (2.1)) are shown in figure 4*a* calculated using the procedure described in §2 [1], with a 2856-term wave function. Figure 4*b* reveals that the radial density–energy relationship is still linear, and the minimum energy corresponds to the peak in the RDF, *D*(*r*_max_). Furthermore, the gradient of the straight line is equal to the total (correlated) energy, e.g. for helium (green dotted line in figure 4*b*) *E*(*r*) = −2.903 724 *D*(*r*). The accuracy of the approximation increases with the accuracy of the wave function used to calculate *E*(*r*) and *D*(*r*). The energy derived using the gradient method is less accurate than the optimized energy for a given matrix size; however, it is still accurate to the *μE*_*h*_ for a modest sized wave function (electronic supplementary material).

The question arises as to whether there is a simple analytical form allowing the energy of a two-electron system to be calculated from the radial density for any nuclear charge *Z*. Helium-like RDFs were calculated for *Z* ∈ [1, 10] and the coordinates at the most probable distance, i.e. (*r*_max_, *D*(*r*_max_)), determined for each system. A variety of functional forms were explored, and it was found that fitting to a cubic polynomial in *D*(*r*_max_) provided very good results. This was assessed by calculating the percentage error in calculating *E*(*r*_max_) using the function fit compared to direct calculation (see electronic supplementary material). The formula obtained by fitting to He, B^3+^ and Ne^8+^ has the following form:

3.7 $$\begin{array}{rl} E(r_{\max}) & =-0.070\;997\;445D(r_{\max})-0.328\;038\;372D{\left(r_{\max}\right)}^{2}-3.412\;073\;050D{\left(r_{\max}\right)}^{3}. \end{array}$$

Given that the total energy corresponds to the gradient of the graph *E*(*r*) against *D*(*r*), we can approximate the fully correlated two-electron energy as follows:

3.8 $$\begin{array}{rl} E & \equiv E[D(r_{\max})]=\frac{E(r_{\max})}{D(r_{\max})}\\ & =-0.070\;997\;445-0.328\;038\;372D(r_{\max})-3.412\;073\;050D{\left(r_{\max}\right)}^{2}. \end{array}$$

Using the energy functional, equation (3.8) provides a good approximation to the total energy of a system. The predicted energies are accurate to at least the *mE*_*h*_, with the exception of the hydride ion as shown in table 1.

**Table 1.** The ground state energy of helium-like systems determined using equation (3.8) derived using a 2856-term wave function. The accuracy of the energy is indicated by the number of digits provided. Note *Z* = 2, 5 and 10 were used in the fitting.

	*E*/*E*_*h*_	
*Z*	optimized energy	equation (3.8)
1	−0.527 751 016 54	−0.52
2	−2.903 724 377 03	−2.903 724
3	−7.279 913 412 66	−7.279
4	−13.655 566 238 42	−13.655
5	−22.030 971 580	−22.030 971
6	−32.406 246 601 8	−32.406
7	−44.781 445 148 77	−44.781
8	−59.156 595 122	−59.156
9	−75.531 712 363 95	−75.531
10	−93.906 806 515	−93.906 806

#### 3.2.2. The 2 ^3^S state of helium-like systems

The RDFs for the 2 ^3^S state of helium-like systems, *Z* ∈ [2, 10] with infinite nuclear mass, were calculated using the procedure described in §2 with a 2600-term wave function [1]. The triplet state RDF contains two peaks, corresponding to, within an orbital picture, the most probable distance of the 1s electron from the nucleus, *r*_max1_, and the most probable distance of the 2s electron from the nucleus, *r*_max2_. As in the previous section, the shape of the RDF, *D*(*r*), and the radial energy, *E*(*r*), as a function of radial coordinate, *r*, mirror each other and result in a straight line in the radial energy–density space. The gradient of the line corresponds to the triplet state energy of the corresponding 2 ^3^S state of the helium-like atom. The energy obtained from the gradient is slightly less accurate than the optimized energy as shown in table 2. The accuracy is dependent on the quality of the wave function, and the loss of accuracy is similar to the loss obtained when calculating the nucleus–electron cusp.

**Table 2.** The 2 ^3^S state energy of helium-like systems determined by (i) calculating the gradient of the line in the radial–energy density space and (ii) using (3.9) derived using a 2600-term wave function and fitting to the data for *Z* = 2, 5, 7 and 10, where the accuracy of the approximation is indicated by the number of digits provided and there is uncertainty in the last digit of the optimized energy.

	*E*/*E*_*h*_		
*Z*	optimized energy	gradient energy	equation (3.9)
2	−2.175 229 378 23	−2.175 229 37	−2.175 229 37
3	−5.110 727 372 57	−5.110 727 372	−5.110
4	−9.297 166 589 777	−9.297 166 589 7	−9.297
5	−14.733 897 348 81	−14.733 897 348 8	−14.733 897 34
6	−21.420 755 902 30	−21.420 755 902	−21.420 7
7	−29.357 681 737 49	−29.357 681 737	−29.357 681 7
8	−38.544 647 320 085	−38.544 647 320	−38.544
9	−48.981 638 329 518	−48.981 638 32	−48.981 638 3
10	−60.668 646 584 073	−60.668 646 58	−60.668 646 5

As for the ground state, a simple analytical form allowing the triplet state energy of a two-electron atom to be calculated from the radial density for any nuclear charge *Z* was determined. The coordinates at the most probable distances, i.e. (*r*_max1_, *D*(*r*_max1_)) and (*r*_max2_, *D*(*r*_max2_)), were determined for each helium-like system, *Z* ∈ [2, 10]. A variety of functional forms were explored for the function passing through (*E*(*r*_max1_), *D*(*r*_max1_)) and for the function passing through (*E*(*r*_max2_), *D*(*r*_max2_)). For the simple polynomials considered, it was found that the fitting using the *r*_max1_ data was slightly superior. Using data generated using a 2600-term wave function, with *E*(*r*_max1_) and *D*(*r*_max1_) calculated with a step-size of 0.0004 bohr and fitting to a function of the form *E*(*r*_max1_) = *a* + *b D*(*r*_max1_) + *c D*(*r*_max1_)^2^ + *d D*(*r*_max1_)^3^ using a training set of *Z* = 2, 5, 7, 10 provided very good results. The optimum function obtained for the energy, where *E* = *E*(*r*_max1_)/*D*(*r*_max1_), has the following form:

3.9 $$\begin{array}{rl} E & =0.002\;090\;022D{\left(r_{\max1}\right)}^{-1}-0.006\;686\;393+0.114\;390\;966D(r_{\max1})-7.226\;958\;296D{\left(r_{\max1}\right)}^{2}. \end{array}$$

The energies obtained using this function are provided in table 2 and demonstrate that the function is capable of predicting triplet state helium-like energies accurate to the *mE*_*h*_.

## 4. Conclusion

We have shown that a linear relationship exists between the radial energy *E*(*r*) and the radial density *D*(*r*), regardless of the number of peaks in the RDF. Plotting this linear relationship in the density–energy space and determining the gradient provides the total energy of the system, *E*, for the atomic systems considered. Given that *D*(0) = *E*(0) = 0, only a single point (*D*(*r*), *E*(*r*)) with *r* ≠ 0 is required to determine the gradient and hence total energy of the system. In the case of one-electron ground and excited state systems, simple, exact and analytical expressions are derived for the total energy in terms of the radial density at the most probable distance(s) in the RDF for the desired state. For two-electron atomic systems, it was found that a quadratic in *D*(*r*_max_) was sufficient to provide fully correlated two-electron energies, for both the singlet and triplet state, accurate to the *mE*_*h*_ for *Z* > 1. The accuracy of the formulae are limited only by the accuracy of the wave functions used to calculate the values of *E*(*r*_max_) and *D*(*r*_max_) utilized in the fitting and *D*(*r*_max_) for the system of interest. Given that these formulae for the calculation of the total energy are dependent only on the value of the radial density at the most probable distance in the RDF, this work holds promise. We plan to explore these radial energy–density relationships further for many-electron systems with *N* > 2.

## Data Availability

The wave function for each of the helium-like systems, *Z* = 1 to 10 for the singlet state and *Z* = 2 to 10 for the triplet state, along with all the radial energy and radial density data presented in the article, and additional computational details are provided in electronic supplementary material [24].
